# Brain Vascular Imaging Techniques

**DOI:** 10.3390/ijms18010070

**Published:** 2016-12-30

**Authors:** Bàrbara Laviña

**Affiliations:** Department of Immunology, Genetics and Pathology, Rudbeck Laboratory, Uppsala University, 75185 Uppsala, Sweden; barbara.lavina-siemsen@igp.uu.se; Tel.: +46-018-471-4504

**Keywords:** brain vascular disorders, imaging, vascular biology, brain atlas, animal models, molecular imaging, computed tomography (CT), positron emission tomography (PET), magnetic resonance angiography (MRI), photoacoustic imaging (PAI), magnetic resonance angiography (MRA)

## Abstract

Recent major improvements in a number of imaging techniques now allow for the study of the brain in ways that could not be considered previously. Researchers today have well-developed tools to specifically examine the dynamic nature of the blood vessels in the brain during development and adulthood; as well as to observe the vascular responses in disease situations in vivo. This review offers a concise summary and brief historical reference of different imaging techniques and how these tools can be applied to study the brain vasculature and the blood-brain barrier integrity in both healthy and disease states. Moreover, it offers an overview on available transgenic animal models to study vascular biology and a description of useful online brain atlases.

## 1. Introduction

The brain is one of the most sophisticated and complex organs generated by evolution and its impressive anatomical composition and its entangled functionality has been admired for thousands of years. Although early civilizations lacked adequate means to obtain knowledge about the nervous system, the ancient Egyptians in the 17th century Before Common Era (BCE) wrote the earliest recorded reference to the brain in the Edwin Smith papyrus [1]. It was not until the 5th century BCE that the concept of the nervous system appeared [2]. In the 1600s, William Harvey proved the theory of blood circulation in *De Motu Cordis* [3], although the first descriptions of the pulmonary circulation by Ibn al-Nafis date back to the 16th century BCE [4]. The interest in dynamics stimulated the study of angiology and neuroanatomy and, in 1664, Thomas Willis published *Cerebri anatome*, a text on the brain that was a groundbreaking work for neuroscience and remained very influential for the next two centuries [5,6].

In 1882, Angelo Mosso invented the first neuroimaging technique, called “human circulated balance” that could non-invasively measure the redistribution of blood during emotional and intellectual activity [7]. Nevertheless, the origin of structural imaging was the X-ray, discovered by Roentgen in 1895 [8]. Shortly after, Haschek and Lindenthal produced radiographs of blood vessels by injecting opaque solution into cadavers; however, it was not until 1927 that Egas Moniz performed the first cerebral angiography in humans [9]. Since then, key events and major technological innovations in physics, mathematics, computing and clinical imaging have promoted the development of at least the following techniques: (1) computed tomography (CT), for which Hounsfield and Cormack were awarded the Nobel Prize in 1979; (2) positron emission tomography (PET) [10]; and (3) magnetic resonance imaging (MRI), for which Lauterbur and Mansfield were awarded the Nobel Prize in 2003 and magnetic resonance angiography (MRA). The abovementioned techniques together with other imaging modalities, including digital subtraction angiography (DSA), photoacoustic imaging (PAI) and trans-cranial doppler (TCD), have contributed to the knowledge of the brain vasculature, promoting and improving our understanding of the complexity of the central nervous system (CNS).

The strategies that scientists have adopted for studying the brain have varied over the years as new techniques and methods have been developed. Elucidating the composition and functions of the brain is one of the most challenging areas of research. Characterizing the structure of the brain at high resolution is crucial for understanding its functions and dysfunction. Neuroimaging, the process of producing images of the structure or activity of the brain, is becoming an increasingly important tool in both research and clinical care, tremendously helping our understanding of brain morphology and physiology in healthy and disease states.

The aim of this review is to give a short overview of the most important recent scientific advances achieved using imaging techniques, with particular focus on their relevance to the field of brain vascular imaging and including a section on molecular imaging of the blood-brain barrier (BBB). A special focus on animal models is also included, as their use is motivated by a desire to better understand human diseases. Moreover, a summary of available online brain atlases for both human and animal models and the way these approaches contribute to a better understanding of the brain on multiple levels are also highlighted.

## 2. Computed Tomography (CT)

On October 1, 1971, in London, England, CT imaging performed by Godfrey Hounsfield and James Ambrose produced the first scan of a patient with a cerebral cyst. The image proved it was possible to produce non-superimposed images of an object slice [11].

Since that time, CT has been improved by a number of important technological advancements, leading to the current ability to acquire thousands of thin-slice images with voxel isotropy in a few seconds with a reduced radiation dose [12,13]. CT angiography (CTA) has gained the most benefit from such evolution in terms of improved diagnostic performance and broadened clinical indications [14,15]. Color-coded CT angiography, a new method of displaying dynamic cerebral CT angiography, provides important additional information on cerebral hemodynamics, including specifically differentiation between antegrade and retrograde flow [16].

Micro-scale computed tomography (microCT) [17,18] and nano-computed tomography (nanoCT) [19] are high-resolution cross-sectional imaging techniques and are essential tools for phenotyping and for elucidating diseases and their therapies. Compared to other imaging methods, the strengths of microCT and nanoCT lay with their high-resolution scanning efficiency, velocity and relatively low cost. Additionally, it is a structural imaging modality that provides a high-resolution volumetric representation of vascular structures in brains of rodents [20,21,22,23,24] as well as measurements of cerebral blood volume (CBV) [25].

Recent applications of microCT to the mouse brain vasculature include the in situ analysis of adult brains using iodine-based contrast [26], the evaluation of animal models of cerebral cavernous malformations (CCM) [27] and the imaging of brain tumors in live mice [28].

Perfusion CT is a relatively new imaging technique that allows rapid qualitative and quantitative evaluation of cerebral vascular physiology and hemodynamics including measurement of cerebral blood flow (CBF) and CBV. It involves the sequential acquisition of cerebral CT images performed during the intravenous administration of contrast material [29]. It is an alternative imaging modality with several clinical indications including stroke [30], head trauma [31] and brain tumors [32]. Published data have suggested that perfusion CT might be comparable to MRI [33,34]. Moreover, perfusion CT has been used to evaluate possible clinical benefits of pharmacology therapy in early stroke onset [35], as well as to predict survival in in high-grade gliomas [36].

## 3. Positron Emission Tomography (PET) and Single Photon Emission Computed Tomography (SPECT)

The development of CT soon led to other imaging techniques, including PET [37,38], which involves injecting rapidly decaying radioactive substances into the bloodstream. The first simple PET scanner able to detect brain tumors was made in the early 1950s by Bromwell and Sweet and by 1975, more sophisticated types of PET, which could measure blood flow, were developed [39,40,41].

The first PET image of a rat brain using a clinical PET scanner was performed in 1991 [42] and the initial dedicated small PET scanner was introduced several years later in 1995 [43]. The recent development of a high-resolution small animal PET scanner suitable for imaging the mouse brain will quickly improve the imaging quality of brain analysis [44].

PET functional imaging is most useful in combination with anatomical imaging and hybrid imaging systems such as PET/MRI [45,46] and PET/CT [47,48]. Multimodality imaging PET/CT and PET/MRI provide advantages in the imaging evaluation of patients with a variety of diseases [49]. A PET/CT scanner has the ability to improve image quality and image accuracy of PET images, enhancing lesion identification and localization, which affects clinical decision making and thereby improves patient management [50,51,52]. PET/MRI combination offers functional structural and metabolic data, which can potentially contribute to more accurate diagnoses and ultimately affect patient survival. The advantages and drawbacks of PET/CT are discussed in [53,54].

Multimodality imaging is nowadays available in the clinical practice and for small animals [55]. Rodent SPECT/CT has been used to assess in vivo CBF and blood-brain barrier (BBB) disruption after focal cerebral ischemia [56] and the recently developed trimodal PET/SPECT/CT scanner for small animals [57] has allowed for new insights into brain function and the visualization of cerebral ischemia in living rats [58].

PET and single photon emission computed tomography (SPECT) [59,60] are molecular imaging modalities which by the use of specific radioactive tracers, allow visualization and measurement of physiological processes in intact living brains [61].

Recently PET has been used to study hypoxia and inflammation in ischemic stroke [62,63,64,65], to explore the possible relationship between an acute ischemic stroke and Aβ deposition in patients [66], and to noninvasively image VEGFR expression kinetics to analyze post stroke angiogenesis in rats [67]. Moreover, ^11^C-methionine is the most popular tracer used in PET imaging of brain tumors [68], and can predict prognosis in gliomas [69]. Other recent examples of brain vascular disorders imaged utilizing PET or SPECT include thrombosis [70], Alzheimer’s [71] and Moyamoya disease [72].

## 4. Magnetic Resonance Imaging (MRI) and Other Similar Techniques

The basic idea behind the MR phenomenon first appeared in 1946 from discoveries by Bloch and Purcell. It took another 25 years before MRI was applied to medical diagnosis, when the first discoveries concerning the development of the technique to visualize different structures were published [73,74,75]. A great advantage with MRI is that it uses magnetic forces rather than potentially harmful ionizing radiation. Moreover, image quality has advanced to a remarkable extent with scan times decreasing by factors of 10 to 100 since the early 1980s [76,77].

The main concepts required to understand MRI include the classification of the image contrast following its sensitivity to three different parameters: proton density (*ρ*), the spin-lattice or longitudinal relaxation times *T*_1_, and the spin-spin or transverse relaxation times *T*_2_ or *T*_2_*. A proton-density-weighted image is a sequence that is mainly sensitive to *ρ* and *T*_1_- or *T*_2_-weighted images are, respectively, sensitive to *T*_1_ or *T*_2_ relaxation times [78]. Additionally, different contrast agents, which enrich MRI, can be used in the so-called Contrast-Enhanced MRI [79]. Several reviews have recently focused on cerebrovascular MRI, compared to other imaging techniques and described the key developments in the last years [12,80,81,82,83].

These new advances in neuroimaging methods could also be applied as biomarkers [84]. Biomarkers can be used as a diagnostic tool, a prognostic tool, a predictive tool (for predicting response to an intervention), or a substitute for a clinical outcome to measure the response to an intervention (surrogate end point). Some examples of biomarkers based on MR imaging used in acute ischemic stroke are summarized in [85]. Biomarkers based on brain imaging may relate to prognosis in high-grade gliomas [86] and recently, vessel caliber analysis has been proposed as a possible biomarker of tumor response in clinical trials, revised in [87].

The study of hemodynamic alterations in patients with cerebrovascular disease is important to understand the disease, potentially improving diagnostic capabilities and therapeutic planning. There is a demand for noninvasive imaging of cerebrovascular territories; therefore, new emerging techniques for evaluation cerebrovascular hemodynamics and CBF [88], including four-dimensional (4D) flow MRI [89,90,91,92]; 2D phase contrast MRI (PC-MRI) [93,94,95] and magnetic resonance black-blood thrombus imaging technique (MRBTI) [96] are being developed.

### 4.1. Diffusion and Perfusion Weighted MRI.

Diffusion (DWI) and perfusion (PWI) weighted MRI have an increasingly important clinical role (see [97] for a detailed description of the basic principles). The combination of both techniques is especially promising for the early detection and assessment of stroke [98,99]; and for brain tumor characterization [100], as they provide complementary information.

DWI is based on the random movement of water molecules caused by their kinetic energy dissipation, known as Brownian motion, in the presence of magnetic pulses. The apparent diffusion coefficient is a measure that displays the magnitude of diffusion of the water molecules within tissue [101,102]. In the field of brain imaging, DWI has been applied to diagnose and monitor stroke [103,104] and characterize brain tumors [105].

PWI refers to methods that make use of the effect of endogenous or exogenous tracers on the MR images for deriving various hemodynamic parameters offering the potential for measuring brain perfusion in several pathological conditions including stroke [106] and brain tumors [107,108]. Perfusion MRI techniques can be used for quantitative assessment of specific pathophysiologic parameters, more accurate grading of intracranial tumors and may predict survival and patient outcome [109,110].

### 4.2. Susceptibility-Weighted Imaging (SWI)

SWI is an MRI technique that enhances image contrast by using the susceptibility differences between tissues and has become a part of routine brain MRI protocols [111]. The clinical success of SWI arises from its superior sensitivity for detecting small quantities of blood product, its ability to differentiate between arterial and venous vessels, and its ability to differentiate between calcification and blood product. Thus, SWI is nowadays utilized to obtain images of diverse brain vascular disorders including: hemorrhages, traumatic brain injury, stroke, tumors and multiple sclerosis [112,113,114]. The fact that this technique does not provide quantitative measurements, which is an important limitation, is currently overcome by the advancement of new technology such as quantitative susceptibility mapping (QSM) [115] and susceptibility tensor imaging (STI) [116].

### 4.3. Quantitative Susceptibility Mapping (QSM)

QSM [117] is expected to play an increasing role in the clinic as it permits to unambiguously differentiate between calcified and hemorrhagic lesions which permits a differential diagnosis and simultaneously reveals brain anatomy. This technique allows investigating and obtaining valuable information not only on compositional changes in aging brain, but also in numerous neurodegenerative disorders, providing valuable guidance to clinicians during diagnosis.

QSM has recently been applied to monitor CCM disease progression and iron deposition [118,119], intracranial hemorrhages [120], hematoma volume [121] and to differentiate hemorrhages from calcifications [122]. The usefulness of QSM in visualizing the microstructure of the mouse brain at a 10 µm resolution, has been shown by the revealing of detailed structures [123].

### 4.4. Intracranial Vessel Wall Imaging (IVW)

Recently available IVW methods provide the possibility of directly assessing the vessel wall [124,125,126,127], providing a useful diagnostic tool, which may improve patient outcomes by helping treatment choice, in comparison to other invasive and non-invasive methods currently available. IVM is especially challenging due to the small caliber and tortuosity of the intracranial vessels and is an upcoming field of interest to assess intracranial atherosclerotic lesions [128,129,130], intracranial vasculopathies [131,132], cerebrovascular inflammation [133], CNS vasculitis [134], brain arteriovenous malformations [135], Moyamoya disease [136,137], Cerebral aneurysms [138,139,140] and intracranial arterial dissection [141].

### 4.5. MR Angiography (MRA)

MRA is a group of techniques based on MRI to image blood vessels [142,143]. MRA techniques can be divided into two categories: contrast-enhanced and non-contrast enhanced MRA. Since its introduction in 1994 by Prince [144], first-pass contrast-enhanced MRA has seen widespread acceptance and details about techniques and contrast agents are reported in [145,146,147,148,149]. A detailed description of non-contrast MRA techniques and the physical mechanisms underlying each method including their clinical applications can be found in [150] and [151]. In the brain, MRA is used to visualize cerebrovascular territories [152,153], and to evaluate stenosis and occlusions [154], aneurysms [155] and other cerebral malformations [156]. A complete description of MRA in brain vascular disorders is reviewed in [157].

## 5. Digital Subtraction Angiography (DSA)

DSA is an imaging method that permits a distinct visualization of the vasculature in a skeletal or dense soft tissue environment. The introduction of the technique in 1980 provided a method for real-time 2D subtraction imaging, spawning a steady progression of related methods, including 3D [158] and 4D DSA [159] (an outline of some historical milestones and future directions are nicely reviewed in [160,161]).

An overview of the technical principles of DSA can be found in [162,163]. Briefly, the target tissue is initially exposed to X-ray or MRI, to obtain the first set of images; then a contrast agent is administered into the vasculature and additional X-ray or MRI are obtained. The mask, which is the first set of images, is then subtracted from the latter or contrast enhanced images, allowing the visualization of the vascular structure free of the surrounding tissue.

DSA can be utilized to visualize intracranial vascular structures [164], vascular abnormalities such as arteriovenous malformations [165], aneurysms [166], carotid stenosis [167], as well as grading Moyamoya disease [168] and collateral flow in acute middle cerebral artery occlusion [169].

## 6. Trans-Cranial Doppler (TCD)

TCD ultrasound is a specialized technique introduced in 1982 by Rune Aaslid for detecting blood flow in the basal intracerebral arteries [170]. It is a noninvasive technique that involves the use of a low-frequency (≤2 MHz) transducer probe to insonate specific areas of the cranium that are relatively thin. TCD enables users to acquire images of some of the major intracranial vessels through the intact skull and monitor cerebral blood flow (CBF) velocity and vessel pulsatility over extended time periods with a high temporal resolution. [171]. Advanced applications of TCD in neurovascular diseases have been extensively revised in [172,173,174,175].

## 7. Photoacoustic Imaging (PAI)

Photoacoustic imaging (PAI) is also called optoacoustic imaging and is an emerging imaging modality that shows great potential for preclinical research and clinical practice [176]. The method is based on the photoacoustic effect; briefly, the tissue of interest is excited by a pulsed laser and part of the locally absorbed light produces thermal excitation, leading to an expansion of the tissue and subsequent generation of ultrasonic waves. Ultrasonic transducers detect the emitted ultrasonic waves, which are finally converted into images.

The applications in biomedicine of the photoacoustic effect, first reported by Alexander Graham Bell in 1880, began in the 1970s [177] but progressed slowly until the last decade of the 20th century, when many pioneering works demonstrated the photoacoustic effect in optically scattering media and biological tissue, extensively reviewed in [178,179,180].

As a hybrid technique, PAI is based on the acoustic detection of optical absorption from either endogenous chromophores or exogenous contrast agents [181], such as chemical dyes [182], nanoparticles [183,184] and reporter genes [185,186]. PAI is especially useful in visualizing blood vessels in vivo due to the fact that blood hemoglobin and deoxyhemoglobin, have a substantially higher absorption than surrounding tissues and therefore create sufficient endogenous contrast. Over the past decade, the photoacoustic technique has been evolving rapidly, leading to a variety of exciting discoveries and applications such as photoacoustic tomography (PAT) that is cross-sectional or three-dimensional (3D) PAI [187,188,189].

PAT allows visualizing and studying a diverse range of structures: from organelles to whole organs [190] and based on the spatial resolution of the method, PAT is classified into optical-resolution photoacoustic microscopy (OR-PAM) [191], acoustic-resolution photoacoustic microscopy (AR-PAM) [192], photoacoustic computed tomography (PACT) [193,194], and photoacoustic endoscopy (PAE) [195].

PAI has been successfully used in the past few years in small animal models to image the brain [196,197] and determine CBF though the intact skull [198]. Recently, a wearable system has been developed that is capable of providing images of cerebral blood vessels noninvasively [199]. Moreover, PAI is a new strategy to visualize and study several brain disorders [200] including stroke [201,202,203], brain tumors [204,205,206]; cerebral edema [207], epilepsy [208]; traumatic brain injury [209] and inflammation [210].

PAT imaging in patients with brain pathologies is currently not used in clinical practice and is still under development. The main challenge and limitation in humans is the thickness of the skull, but promising recent advances suggest that this technology could be implemented as a clinical device for noninvasive functional brain imaging [184,211,212,213].

## 8. Molecular Imaging of the Blood-Brain Barrier (BBB)

Multiple cell types, which coordinately form the neurovascular unit (NVU), include neurons; vascular cells (endothelial cells (EC), smooth muscle cells and pericytes); and glia (astrocytes, microglia and oligodendroglia), which are key factors to maintaining CNS functions. Within the NVU, the EC form the BBB that limits entry of substances into the brain and maintains the ideal environment for the brain to properly function [214,215].

A detailed molecular atlas of the BBB transport systems and cellular functions, based on available data on protein and RNA expression, as well as physiological measurements from different published investigations, is meticulously provided in this recent review [216].

Dysfunction of the BBB has been shown to be a common denominator in many CNS disease pathogeneses observed in stroke, cerebral edema, Parkinson’s disease, Alzheimer’s disease, seizures, microcephaly and CCMs, among others [214,217,218].

Work in key molecular components of the ECs composing the BBB—including macromolecule transporters such as the glucose transporter GLUT1 [219]; the major facilitator superfamily domain-containing protein 2a (MFSD2a) [220,221]; and tight junction complexes [222], such as Occuldin [223]; and the junctional adhesion molecule C (JAM-C) [224]—and their implication in several brain diseases with BBB dysfunction and altered cerebrovascular integrity have raised the interest in molecular imaging of the BBB.

Dynamic contrast-enhanced MRI (DCE-MRI) is the most widely used imaging method for assessing BBB integrity [225,226,227,228]. It has been used to asses abnormal BBB permeability in several pathological conditions including: traumatic brain injury [229], vascular cognitive impairment [230], multiple sclerosis [231], Alzheimer’s disease [232], brain tumors [233] and stroke [234].

The development of novel state-of-the art neuroimaging and molecular biomarker approaches are key aspects of future investigations addressing whether molecular and imaging biomarkers of BBB dysfunction can serve as reliable prognostic and/or diagnostic tools to predict the development of several CNS disorders [235].

## 9. Transgenic Animal Models for Vascular Biology

Recent advances in imaging techniques [236,237] together with the development of tissue-clearing methods, facilitating volumetric imaging without sectioning [238,239,240], do now provide the possibility, with adequate resolution, to obtain live imaging data from the vasculature in organs and even whole experimental animals during development and disease conditions.

The development of transgenic animal models with fluorescent markers for specific proteins or specific vascular cell types are necessary to fully benefit from the newly available microscope techniques.

So far, zebrafish has been the most suitable animal model to perform in vivo live imaging experiments of the vascular system. Therefore, several transgenic lines, which express different kinds of fluorescent probes in vascular cell types, are currently available and have extensively contributed to improving our knowledge of key vascular processes such as lumen formation, angiogenic sprouting, remodeling, cell proliferation and circulation of blood cells, reviewed in [241,242].

Although the establishment of fluorescent vascular reporters in mice models to study vascular biology has been slower, it is in fact becoming more relevant. Some transgenic mouse lines are already available and label different cell types including: EC [243,244,245,246,247,248,249,250,251,252]; lymphatic endothelial cells (LEC) [253,254,255,256,257], reviewed in [258] and pericytes [259,260,261]. While the above referenced transgenic lines are useful reporters for the major vascular cell types, other transgenic lines have been developed to visualize various organelles or subcellular structures [262,263,264,265].

The effort to develop new tools such as florescent reporter mice is not only performed by single scientific groups but also by joined projects. One example is the Gene Expression Nervous System Atlas (GENSAT) project, which is mapping the expression pattern of genes in the CNS and has created several mouse reporters, expressing enhanced green fluorescent protein (EGFP) and td-Tomato, to investigate the distinct gene expression patterns [266] (www.gensat.com). Moreover, other interesting fluorescent reporter mice are also available in the Jackson Laboratory (www.jax.org).

## 10. Brain Atlases

Another useful tool generated using some of the imaging methods mentioned in this review are brain atlases [267]. Brain atlases are applicable in in all areas of neuroscience including research, education and clinical applications. An enormous amount of data is produced every day in laboratories worldwide regarding brain mapping, and there are many initiatives to make these data available through public databases. At present, brain atlases are printed, electronic, web-based and some are even available on mobile platforms [268].

The recent technical advances in areas such as sample preparation, optical techniques, quantitative 2D and 3D imaging analysis and high-performance computing, have relevantly contributed to the development of new brain mapping approaches (reviewed in [269]). The latest news about the worldwide brain research initiatives can be found in [270].

Following the publication of the mouse [271], adult [272] and prenatal [273] human brain gene expression atlases in recent years, a high-resolution transcriptional atlas of pre- and post-natal brain development for the rhesus monkey has now become available [274].

Table 1 gives examples of some of the most relevant web-based brain atlases in human, rodents and other species [271,272,274,275,276,277,278,279,280,281,282,283,284,285,286,287,288,289,290,291,292,293].

## 11. Conclusions

Imaging is becoming an increasingly important tool in research and clinical care. A range of imaging techniques now provides unprecedented capacity to visualize the structure of the brain, including the vasculature, from the level of individual molecules and encompassing the whole brain. Most imaging methods are noninvasive and allow monitoring dynamic processes over time. Imaging enables researchers to identify brain vascular abnormalities, understand disease pathways, evaluate blood-brain barrier (BBB) integrity, recognize and diagnose pathologies and determine efficacy of different therapies and treatments. Each technique has strengths and weaknesses relating to cost, availability, temporal and spatial resolution and risk factors, thus improving our knowledge and ensuring the innovation of such tools will be beneficial in the near future.

The imaging techniques described in this review, alone and in combination, contribute to transforming and improving our understanding of how the brain functions in health and disease.

## Figures and Tables

**Table 1:** Examples of available online brain atlases.

Specie	Atlas Name	Comments	Link	Selected References
Human	The Human Brain Atlas at Michigan State University	MRI from one specimen + myelin and cell bodies staining	https://msu.edu/~brains/brains/human/index.html	Tool developed by Sudheimer, K.D.; Winn, B.M.; Kerndt, G.M.; Shoaps, J.M.; Davis, K.K.; Fobbs Jr., A.J.; Johnson; J.I.
	Allen Brain Atlas	MRI, in situ hybridization, microarray	http://human.brain-map.org/	[272,275,276]
	Scalable Brain Atlas	Brain atlas templates for different species	https://scalablebrainatlas.incf.org/main/index.php	[277]
	Atlas of the Human Brain	Macroscopic and microscopic levels	http://www.thehumanbrain.info/	[293]
	Human Connectome Project	MRI-based imaging modalities to measure brain architecture, function and connectivity	http://www.humanconnectomeproject.org/	[278,279,280,281]
	BigBrain	Cell bodies’ staining	https://bigbrain.loris.ca/main.php	[282]
	Jubrain	Probabilistic maps based on post mortem brains	https://www.jubrain.fz-juelich.de/apps/cytoviewer/cytoviewer-main.php	[283]
	The Whole Brain Atlas	Contains atlas from different cerebrovascular diseases	http://www.med.harvard.edu/aanlib/home.html	[284]
	Human Brainnetome Atlas	Structural and connectivity features	http://atlas.brainnetome.org/	[285]
	Brain Development Atlases (Imperial College London)	Different atlas datasets are available (adult, pediatric and neonatal)	http://brain-development.org/brain-atlases/	[286,287,288]
	FSL Atlas	Library of analysis tools for FMRI, MRI and DTI brain imaging data	http://fsl.fmrib.ox.ac.uk/fsl/fslwiki/	[289]
Rodents	Allen Brain Atlas	Includes developing brain, spinal cord and connectivity brain atlas	http://mouse.brain-map.org/	[271,276]
	Mouse Atlas Project	Contains also a neonatal brain atlas	http://map.loni.usc.edu/	[290]
	The Mouse Brain Library	Includes developing mouse brain atlas	http://www.mbl.org/	Tool developed by Williams, R.W. and colleagues
Others	Zebrafish Brain Atlas	Includes information about the neuroanatomy of the developing zebrafish brain	http://zebrafishbrain.org/	[291]
	Blueprint Atlas (Rhesus macaque)	Gene expression data, neuroanatomical data	http://www.blueprintnhpatlas.org/	[274]
	Virtual Fly Brain (Drosophila melanogaster)	Neural anatomy and imaging data	http://www.virtualflybrain.org/	[292]

## Abbreviations

BCE	Before Common Era
CNS	central nervous system
CT	computed tomography
MRI	magnetic resonance imaging
PET	positron emission tomography
SPECT	single photon emission computed tomography
CTA	computed tomography angiography
MicroCT	micro-computed tomography
NanoCT	nano-computed tomography
CCM	cerebral cavernous malformations
QSM	quantitative susceptibility mapping
STI	susceptibility tensor imaging
SWI	susceptibility-weighted imaging
4D	four-dimensional
IVW	intracranial vessel wall imaging
PAI	photoacoustic imaging
PAT	photoacoustic tomography
3D	three-dimensional
2D	two-dimensional
EC	endothelial cells
LEC	lymphatic endothelial cells
MRBTI	magnetic resonance black-blood thrombus imaging technique
NVU	neurovascular unit
DCE-MRI	dynamic contrast-enhanced MRI
BBB	blood-brain barrier
CBV	cerebral blood volume
TCD	trans-cranial doppler
PC-MRI	phase contrast MRI
MRA	magnetic resonance angiography
DSA	digital subtraction angiography
DWI	diffusion weighted MRI
PWI	perfusion weighted MRI
EGFP	Enhanced green fluorescent protein
